# Factors affecting cell-free DNA fetal fraction: statistical analysis of 13,661 maternal plasmas for non-invasive prenatal screening

**DOI:** 10.1186/s40246-019-0244-0

**Published:** 2019-12-04

**Authors:** Yaping Hou, Jiexia Yang, Yiming Qi, Fangfang Guo, Haishan Peng, Dongmei Wang, Yixia Wang, Xiaohui Luo, Yi Li, Aihua Yin

**Affiliations:** 1 Medical Genetic Centre, Guangdong Women and Children Hospital, Guangzhou, 511400 Guangdong China; 2 Maternal and Children Metabolic-Genetic Key Laboratory, Guangdong Women and Children Hospital, Guangzhou, 511400 Guangdong China

**Keywords:** Cell-free fetal DNA (cffDNA), Fetal fraction, Gestational age, Maternal BMI, Maternal age

## Abstract

**Background:**

The identification of cell-free fetal DNA (cffDNA) facilitated non-invasive prenatal screening (NIPS) through analysis of cffDNA in maternal plasma. However, challenges regarding its clinical implementation become apparent. Factors affecting fetal fraction should be clarified to guide its clinical application.

**Results:**

A total of 13,661 pregnant subjects with singleton pregnancies who undertook NIPS were included in the study. Relationship of gestational age, maternal BMI, and maternal age with the cffDNA fetal fraction in maternal plasmas for NIPS was investigated. Compared with 13 weeks (12.74%) and 14–18 weeks group (12.73%), the fetal fraction in gestational ages of 19–23 weeks, 24–28 weeks, and more than 29 weeks groups significantly increased to 13.11%, 16.14%, and 21.17%, respectively (*P* < 0.01). Compared with fetal fraction of 14.54% in the maternal BMI group of < 18.5 kg/m^2^, the percentage of fetal fraction in the group of 18.5–24.9 kg/m^2^ (13.37%), 25–29.9 kg/m^2^ (12.20%), 30–34.9 kg/m^2^ (11.32%), and 35–39.9 kg/m^2^ (11.57%) decreased significantly (*P* < 0.01). Compared with the fetal fraction of 14.38% in the group of 18–24 years old, the fetal fraction in the maternal age group of 25–29 years old group (13.98%) (*P* < 0.05), 30–34 years old group (13.18%) (*P* < 0.01), 35–39 years old group (12.34%) (*P* < 0.01), and ≥ 40 years old (11.90%) group (*P* < 0.01) decreased significantly.

**Conclusions:**

The percentage of fetal fraction significantly increased with increase of gestational age. Decreased fetal fraction with increasing maternal BMI was found. Maternal age was also negatively related to the fetal fraction.

## Background

In 1997, cell-free fetal DNA (cffDNA) was first discovered in maternal plasma and serum samples by identifying Y-specific DNA fragments [1]. cffDNA is thought to come from apoptotic trophoblastic cells, and it is mainly derived from the placental origin [2]. cffDNA is a very small fragment (less than 200 base pairs), which comprises fragments of DNA that are shorter on average than maternal cell-free DNA [3]. Generally, its concentration is about 10% of total cell-free DNA (cfDNA) in maternal plasma [4]. It presents in the maternal circulation from early in pregnancy [5] and is rapidly cleared from maternal blood following 2 h after delivery [6, 7]. Therefore, cffDNA is a useful potential source of fetal genetic material for noninvasive prenatal screening (NIPS). Nowadays, cffDNA, as a promising molecular biomarker, has been applied in various aspects of obstetrical research, notably in prenatal diagnosis and complicated pregnancies. One example is to detect the presence of fetal aneuploidies noninvasively from the plasma fraction of maternal blood samples [8–13]. However, with the deep understanding and wide application of the technology, challenges regarding its clinical implementation become apparent. Some of these challenges include test failures, false-positive and false-negative results, limitations in positive predictive value in low-prevalence populations, and potential maternal health implications of abnormal results.

With the increasing use of cffDNA for aneuploidy screening in clinical practice by NIPS, the proportion of cffDNA belonging to the fetus, known as the fetal fraction (FF), is considered an important component to ensure the accuracy of NIPS [14]. Fetal fraction is known to be affected by gestational age, maternal weight, placental size and function, and other various factors. Study showed that fetal fraction was increased with the gestational age of the fetus [4, 15]. Previous studies suggested that maternal weight or maternal BMI has a strong correlation with cfDNA: as maternal weight or maternal BMI increases, fetal fraction decreases [16–19]. However, factors that are known to influence the fetal fraction of cfDNA have not reached a widespread consensus among various studies, mainly because the sample used is too small to attain reliable statistical analysis. Therefore, with the aim of enriching and providing some meaningful clinical data from a large number of clinical cases, there was retrospective analysis by evaluating the association between gestational age, maternal BMI, maternal age, and cffDNA fetal fraction from 13,661 maternal plasmas for NIPS in the present study.

## Methods

### Sample collection

The study set included 13,661 maternal plasma samples drawn between 20 July 2014 and 31 December 2016 from women with singleton pregnancies who undertook NIPS at Guangdong Women and Children Hospital. All the subjects in the study were classified into different groups based on gestational age, body mass index (BMI) (weight and height were used to calculate BMI), and maternal age. Women were offered NIPS due to an increased risk of a chromosomal abnormality based on maternal age, ultrasound evaluation, or history of aneuploidy. Women were offered genetic counseling before and after NIPS testing and results. Those who had a multiple pregnancy were excluded from the study. All pregnant women gave written informed consent to participate in the study which was approved by the Ethics Committee of Guangdong Women and Children Hospital. All research was performed in accordance with the relevant guidelines and regulations.

### Sequencing analysis of maternal plasma DNA

The cffDNA was isolated from maternal peripheral blood samples which were collected into Cell-Free DNA BCT™ tubes (Streck) and sent to the clinical laboratory within 72 h after collection. The cffDNA was isolated by a double centrifugation procedure and stored frozen at − 70 °C until further processing. Cell-free DNA was extracted from 500 μL of maternal plasma with the QIAamp DSP DNA Blood Mini Kit (Qiagen) following the blood and body fluid protocol. Then, the DNA library construction was prepared from 50 μL of extracted DNA solution from maternal plasma according to the manufacturer’s instructions of the Ion Plus Fragment Library Kit (Life Technologies). After quantification on the 7500 real-time PCR platform (Life Technologies), the libraries from 15 different samples were pooled and semiconductor-sequenced on an Ion Proton sequencer at 400 flows according to the manufacturer’s instructions (Life Technologies).

Finally, samples were sequenced, analyzed blindly, and aligned to the UCSC hg19 version of the human genome using Bowtie version 2. *Z*-scores were calculated for the targeted chromosomes 13, 18, and 21 as described, and classification was based upon a standard normal transformed cutoff value of *z* = 3 for chromosome 21 and *z* = 3.95 for chromosomes 18 and 13 [20]. The fetal fraction in pregnancies with male fetuses was calculated from reads proportion of Y chromosome sequences in maternal plasma. For pregnancy with a female fetus, the fetal fraction could be estimated using the length distribution of cffDNA. Investigation on the method of fetal fraction estimation in maternal plasma DNA has been set as described in detail by previous reports [21, 22]. Results were expressed as “positive” or “negative” when the metric criteria (total count of reads should be ≥ 9 million, no amplification bias, and the estimated fetal fraction ≥ 4%) were fulfilled and “no-result” if they were not. Results of NIPS were presented within 2 weeks after the sample was received.

### Statistical analysis

Statistical analyses were performed by using a commercially available software package SPSS 13.0. All data were expressed as means ± standard deviation (M ± SD). Statistical significant difference was tested by one-way ANOVA with post hoc tests. Differences were considered statistically significant for *P* < 0.05 and marked by different symbols in the figures. The risk probability of fetal chromosomal aneuploidy disease (chromosome 21, 18, or 13) detected by high-throughput sequencing was statistically analyzed by the special non-invasive prenatal data analysis management system software based on the binary hypothesis *Z*-score evaluation criteria.

## Results

### Dataset summary of the studied population

A total of 13,661 subjects with singleton pregnancy undergoing NIPS at Guangdong Women and Children hospital during the period of July 20, 2014, to December 31, 2016, were used for the present study. The detailed demographic and pregnancy characteristics of these cases are summarized in Table 1. For the 13,661 subjects, the lowest fetal fraction was 4.01% and the highest was 49.12%, with a statistical median fetal fraction of 12.65%. Considering the gestational age, the minimum was only 11 weeks and the largest was 37 weeks, with the median reported gestational age at sampling for NIPS of 17 weeks. The minimum and largest BMI of subjects in the group was found to be 14.00 kg/m^2^ and 45.00 kg/m^2^, respectively, with the median BMI of subjects in the group of 22.00 kg/m^2^. When taking maternal age into consideration, the range was found between 18 and 50 years, with a median of 32 years old (Table 1).

**Table 1:** Summary on fetal fraction and maternal characteristics of 13,661 women with singleton pregnancy undergoing NIPS in the studied population

	Median	Std. deviation	Minimum	Maximum
Fetal fraction (%)	12.65	4.88	4.01	49.12
Gestational age (weeks)	17.00	3.92	11.00	37.00
Maternal BMI (kg/m^2^)	22.00	3.18	14.00	45.00
Maternal age (years)	32.00	5.50	18.00	50.00

*BMI* body mass index

### Effect of gestational age on the fetal fraction

**Fig. 1:**
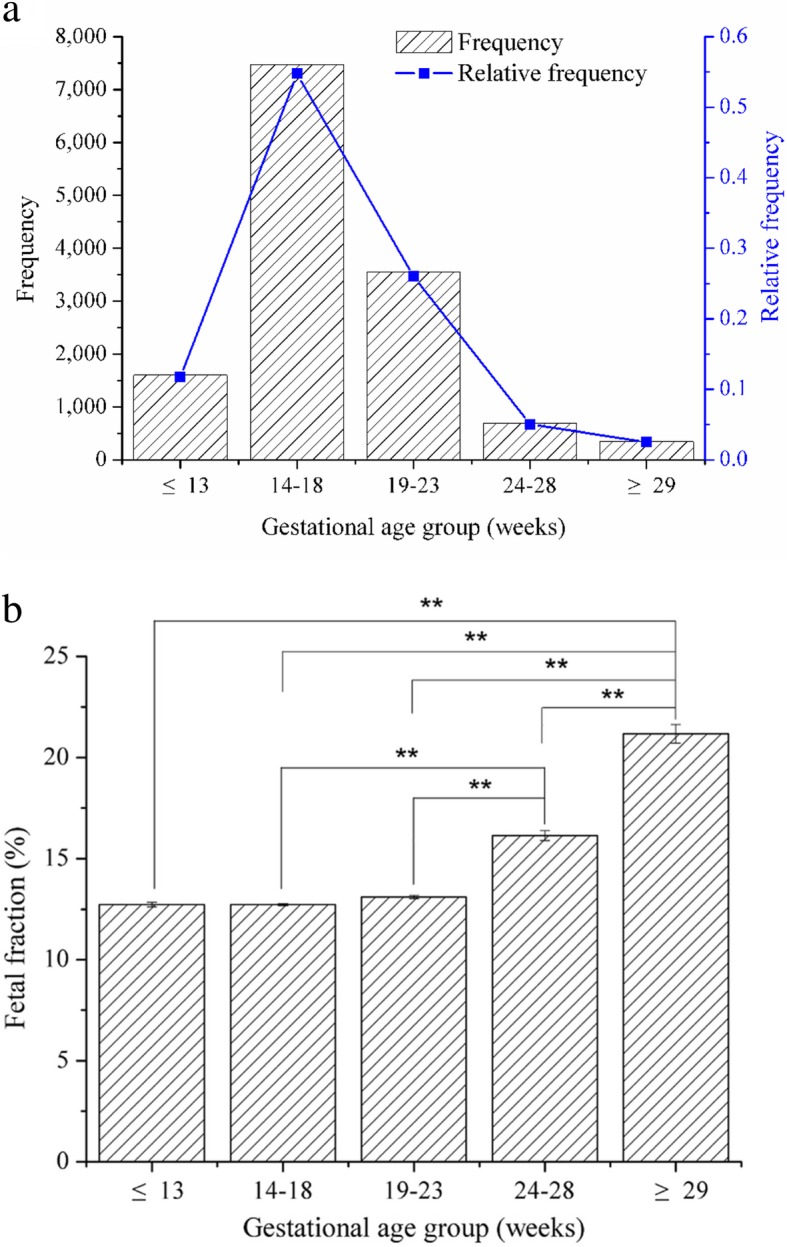
Effects of gestational age on the percentage of fetal fraction. **a** Distribution of gestational age across all 13,661 subjects in this study. **b** Effects of gestational age on the percentage of fetal fraction between different groups. Two asterisks represent the significant difference at *P* < 0.01 level

In the present study, five different groups were classified based on the gestational age, with lower than 13 weeks, 14–18 weeks, 19–23 weeks, 24–28 weeks, and more than 29 weeks groups, respectively. Among the 13,661 cases, 1601samples belong to lower than 13 weeks group (relative frequency of 11.72%). A total of 7478 reported gestational age at sampling for NIPS which was found between 14 and 18 weeks, with the relative frequency of 54.74%. A total of 3550 samples were found between 19 and 23 weeks (relative frequency of 25.99%). The remaining 690 were between the group of 24–28 weeks (5.05%) and 342 samples for more than 29 weeks (2.50%), respectively (Fig. 1a).

Basically, the fetal fraction enhanced with the increase of gestational ages. The mean fetal fraction in lower than 13 weeks and 14–18 weeks groups was 12.74% and 12.73%, respectively. No significant change on the fetal fraction between these two groups was found while doing the statistical analysis (*P* > 0.05). However, compared to these two groups, the fetal fraction in 19–23 weeks, 24–28 weeks, and more than 29 weeks groups increased to be 13.11%, 16.14%, and 21.17%, respectively. Significant difference at *P* < 0.01 level was found on the fetal fraction compared to the above two groups. Moreover, significant difference among these three groups was also found (*P* < 0.01) (Fig. 1b).

### Effect of maternal BMI on the fetal fraction

**Fig. 2:**
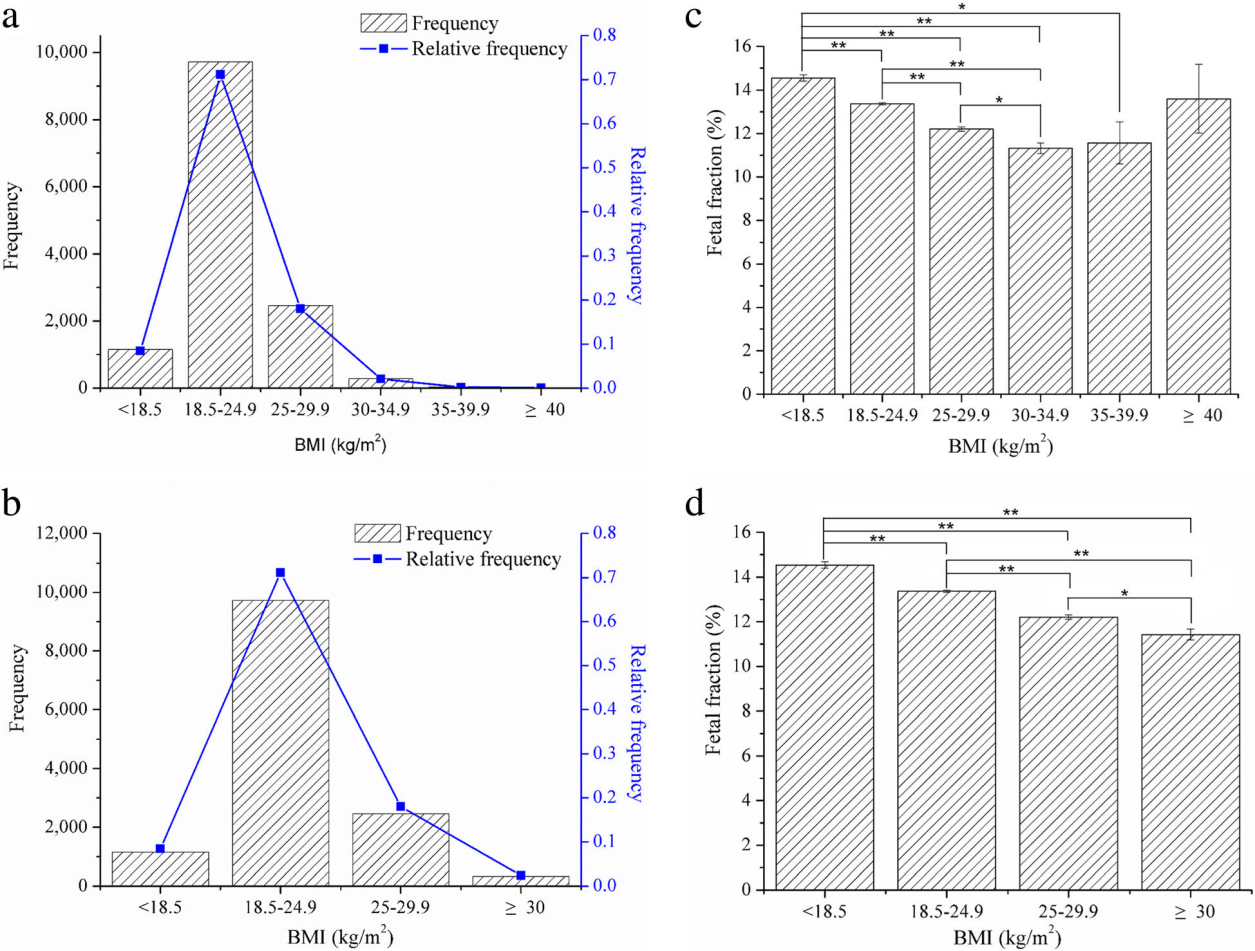
Effects of BMI on the percentage of fetal fraction. **a** Distribution of BMI across all 13,661 subjects in the six groups. **b** Distribution of BMI across all 13,661 subjects in the four groups. **c** Effects of BMI on the percentage of fetal fraction between the six groups. **d** Effects of BMI on the percentage of fetal fraction between the four groups. One asterisk represents the significant difference at *P* < 0.05 level. Two asterisks represent the significant difference at *P* < 0.01 level

Weight and height were used to calculate body mass index (BMI), and the 13,661 subjects were classified into six groups based on the World Health Organization obesity classification system. Results indicated that 71.17% of cases fall within the maternal BMI range of 18.5–24.9 kg/m^2^. Besides, there were 18.00% and 8.43% of cases which belong to the group of 25–29.9 kg/m^2^ and < 18.5 kg/m^2^, respectively (Fig. 2a). On the account of the small size of each group, the three groups 30–34.9 kg/m^2^, 35–39.9 kg/m^2^, and ≥ 40 kg/m^2^ were also grouped into a new group (≥ 30 kg/m^2^). And this new group included 2.40% of the subjects (Fig. 2b).

Results showed a decrease of fetal fraction with increasing maternal BMI (Fig. 2d). Compared with fetal fraction of 14.54% in the group of < 18.5 kg/m^2^, the percentage of fetal fraction in the group of 18.5–24.9 kg/m^2^ (13.37%), 25–29.9 kg/m^2^ (12.20%), 30–34.9 kg/m^2^ (11.32%), and 35–39.9 kg/m^2^ (11.57%) was significantly decreased (*P* < 0.01), respectively. Besides, it was also significantly decreased in the group of 25–29.9 kg/m^2^ (*P* < 0.01) and 30–34.9 kg/m^2^ (*P* < 0.01) when compared with the group of 18.5–24.9 kg/m^2^. Moreover, there was also a significant difference between the groups of 25–29.9 kg/m^2^ and 30–34.9 kg/m^2^ (*P* < 0.05) (Fig. 2c).

### Effect of maternal age on the fetal fraction

**Fig. 3:**
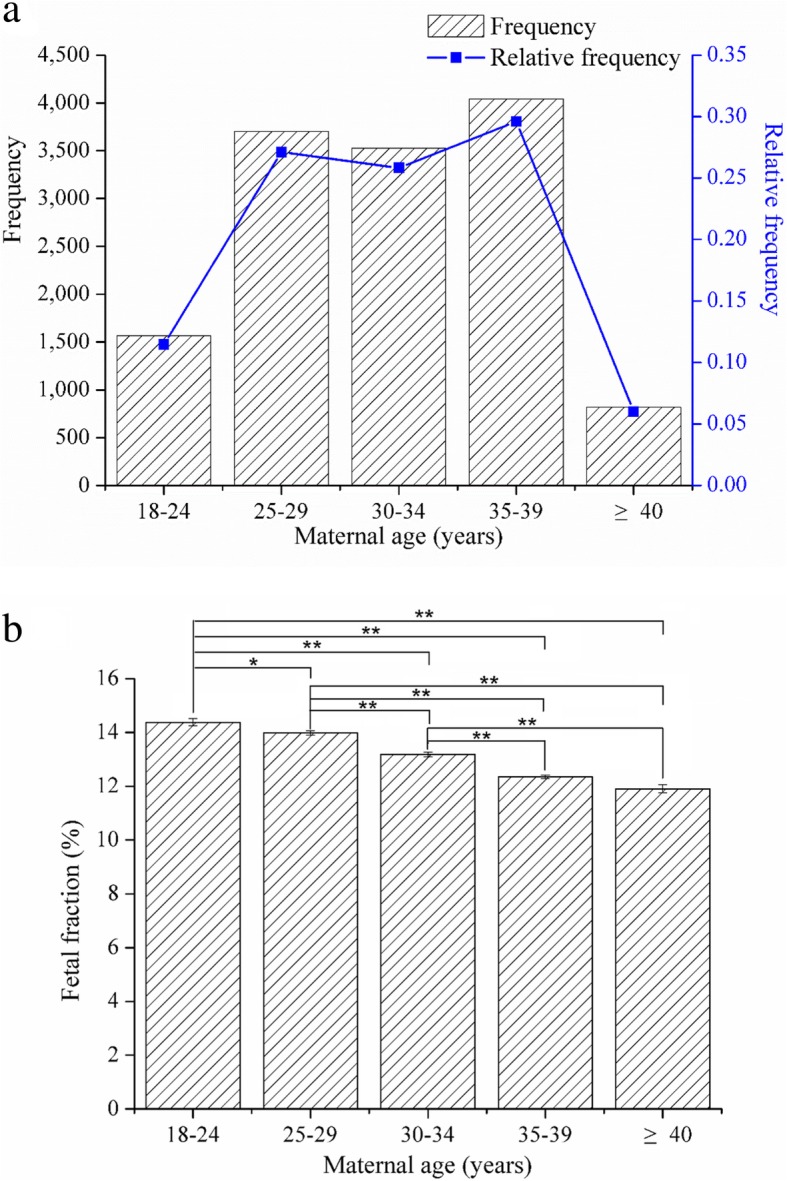
Effects of maternal age on the percentage of fetal fraction. **a** Distribution of maternal age across all 13,661 subjects in this study. **b** Effects of maternal age on the percentage of fetal fraction between different groups. One asterisk represents the significant difference at *P* < 0.05 level. Two asterisks represent the significant difference at *P* < 0.01 level

Most of the subjects belong to the group of 25–29 years old (3704, 27.11%), 30–34 years old (3528, 25.83%), and 35–39 years old (4043, 29.60%). The other small parts were cases in the group of 18–24 years old (1567, 11.47%) and ≥ 40 years old (819, 6.00%) (Fig. 3a). Results indicated that the percentage of fetal fraction significantly decreased with the increase of maternal age (Fig. 3b). Compared with the fetal fraction of 14.38% in the group of 18–24 years old group, the percentage of fetal fraction in the group of 25–29 years old group (13.98%) (*P* < 0.05), 30–34 years old group (13.18%) (*P* < 0.01), 35–39 years old group (12.34%) (*P* < 0.01), and ≥ 40 years old (11.90%) (*P* < 0.01) decreased significantly. Moreover, significant decrease was also found in the group of 30–34 years old (*P* < 0.01), 35–39 years old (*P* < 0.01), or ≥ 40 years old (*P* < 0.01) compared with the group of 25–29 years old. Similarly, the percentage of fetal fraction in either group of 35–39 years old (*P* < 0.01) or ≥ 40 years old (*P* < 0.01) was significantly lower than that in the group of 30–34 years old (Fig. 3b).

## Discussion

This is the large-scale clinical study to evaluate the effects of gestational age, maternal BMI, and maternal age on fetal fraction in maternal plasma undergoing NIPS in a clinical setting. NIPS has been widely used to screen for Down syndrome (trisomy 21), Edward syndrome (trisomy 18), and Patau syndrome (trisomy 13) in the past few years [11, 13, 23], yet large clinical data is still absent, and concerns have been raised about the performance in large-scale clinical practice. To provide the large clinical data in this field, the present study was performed in 13,661 subjects with singleton pregnancy undergoing NIPS at Guangdong Women and Children hospital (Table 1).

Present results suggested that there were some effects of gestational age on the fetal fraction. Results showed that the fetal fraction increased incrementally from 19 and 23 weeks of gestation. In general, there was a greater increase in fetal fraction with the increase in gestational age (Fig. 1b). These findings are similar with previous studies: Wang et al. found that fetal fraction increased incrementally between 10 and 21 weeks of gestation and over this gestational age window [16]. Besides, recent study also reported that the fetal fraction increased tenfold from 20 weeks’ gestation and thought that the rate of increase in fetal fraction with increasing gestational age varies across the duration of the testing period [24]. Therefore, the result of this study showed that gestational age is one of the factors that affect the increase of level of the fetal fraction.

Though fetal fraction was shown to trend positively with gestational age, the strong negative correlation was observed for either fetal fraction or maternal BMI from the present study. In general, results from either six groups or four groups suggested that fetal fraction decreases with maternal BMI (Fig. 2c, d). As we know, obesity is associated with various alterations in the maternal metabolic profile, such as increasing the maternal total blood volume, turnover of adipocytes, white blood cell count, and stromal vascular apoptosis [19, 25]. Therefore, the concentration of circulating maternal DNA in the circulation was increased owing to those changes in obese subjects. In accordance with the previous studies, the decrease tendency of fetal fraction has been attributed to the dilution of a fixed amount of fetal fraction in obese pregnant women [18, 19, 26, 27]. Therefore, for patients with increased BMI, the medical staff should essentially pay attention to the fetal fraction.

Moreover, the present results showed that maternal age also had negative effect on the level of fetal fraction. In the present study, most of the pregnant women were 25–39 years old, and nearly one third of them belong to 35–39 years old (Fig. 3a). Consistent with the previous result [28], the present result from this population showed that the percentage of fetal fraction significantly decreased with increasing maternal age (Fig. 3b). Previous comment has also suggested that the role of maternal age or other factors for aneuploidy may affect the performance of prenatal screening using cfDNA [29]. However, study also suggested that fetal fraction had no correlation with maternal age [30]. Thus, the impact of maternal age on fetal fraction has not reached a consensus and still need further data. However, initial guidelines from all major societies recommended limiting the use of cfDNA screening to those pregnancies which included age 35 years old at the time of delivery, history of a prior aneuploidy, and so on [31]. In China, pregnant women aged 35 years or older usually were suggested to receive invasive prenatal diagnosis directly. However, more and more experts believed that it was not appropriate to regard the age as the only indication for the choice of prenatal diagnosis recently. Therefore, clinicians and counselors should be familiar with the performance of the test used and aware of the effect of maternal condition, such as maternal age and BMI.

This study provides clinically useful data for the utility of NIPS and diagnostic testing. However, due to certain reasons, there are still some limitations and further work should be focused on the present study. One of the limitations is that our study does not evaluate the relationship between fetal fraction and other factors, such as free β-hCG and PAPP-A. Another limitation is the lack of data analysis on the fetal fraction and the positive results. Besides, it is worth noting that our study lacks the clinical data on the other maternal conditions, for instance, the blood glucose and lipid level, and other potential confounding characteristics of the pregnant subjects, such as smoking, intemperance, and dietary habit. Due to the limitations within the present clinical data, other clinical factors, except gestational age, maternal BMI, and maternal age, which influence the fetal fraction, remain a substantial amount for clinical data and further investigation.

## Conclusions

Present study provides clinically meaningful data to determine the effects of gestational age, maternal BMI, and maternal age on fetal fraction in maternal plasma undergoing NIPS. Results from this study indicate that the percentage of fetal fraction significantly enhanced with increasing of gestational age. However, overall, there was strong negative correlation between fetal fraction and maternal BMI maternal age. In conclusion, though it is relatively safer and more efficient, various factors could affect the accuracy of NIPS results. Therefore, NIPS is still a screening test, and comprehensive counseling of pre⁃ and post⁃NIPS should be performed in clinical practice. The clinicians and counselors should be familiar with related guidelines and aware of the complexity of NIPS, especially the specific maternal conditions that may affect the performance of NIPS.

## Data Availability

Please contact author for data requests.
